# Optimal analgesic regimen for total shoulder arthroplasty: a randomized controlled trial and network meta-analysis

**DOI:** 10.1186/s13018-023-04451-8

**Published:** 2024-01-12

**Authors:** Shiye Li, Wenjie Chen, Liang’en Feng, Xu Guo

**Affiliations:** 1 Pain Department of Hezhou People’s Hospital, Hezhou, China; 2 Spine and Orthopaedic Department of Hezhou People’s Hospital, Hezhou, China

**Keywords:** Total shoulder replacement, Liposomal bupivacaine, Local infiltration analgesia, Single-shot interscalene block, Continuous interscalene block, Network meta-analysis

## Abstract

**Objective:**

Clinical approaches to analgesia following total shoulder arthroplasty include liposomal bupivacaine, local infiltration analgesia, single-shot interscalene block, and continuous interscalene block. However, the best method remains contentious. This study conducts a network meta-analysis comparing these four methods, aiming to identify the most effective analgesic approach.

**Methods:**

Randomized controlled trials on analgesic regimens for total shoulder arthroplasty were identified through searches of PUBMED, Cochrane Central Register of Controlled Trials, EMBASE, Web of Science, and Scopus databases, covering their inception through November 2023. Network meta-analysis was performed using STATA 15.1, and the Cochrane Handbook version 5.1.0 risk of bias tool was employed for quality assessment of the literature.

**Results:**

Twelve randomized controlled trials were included, comprising 1537 patients undergoing total shoulder arthroplasty. The interventions compared were ssISB, cISB, LIA, and LB. Regarding the quality of the literature, four studies were deemed low risk, one high risk, and seven moderate risk. The network meta-analysis revealed that in terms of VAS scores in the PACU, the ssISB group was the most effective, followed by cISB and LB, with LIA being the least effective. This pattern continued in VAS scores on the first and second postoperative days. Regarding morphine consumption, the cISB group showed the most significant reduction in the PACU and on the first postoperative day, while the LIA group performed best in total postoperative morphine consumption. The shortest average hospital stay was noted in the cISB group.

**Conclusion:**

The ssISB method excels in controlling early postoperative pain, particularly during the PACU stage and early postoperative period. Additionally, the cISB method is notable for reducing postoperative morphine consumption and shortening average hospital stays. While the LIA method ranks first in reducing total morphine consumption, it is weaker in pain control. The LB method is underwhelming across most assessment parameters. These findings underscore the importance of selecting appropriate analgesic strategies for different postoperative recovery phases and provide valuable insights for clinicians to optimize postoperative pain management. Furthermore, they suggest a need for future research to explore the specific application and effectiveness of these methods in varying clinical contexts.

## Introduction

With the advent of an ageing society, the incidence of shoulder osteoarthritis is escalating, leading to pain and reduced functional activity, often culminating in end-stage joint disease [1]. Joint replacement surgery seems to be the optimal solution to this dilemma. Over the past two decades, a study in the USA revealed a twofold increase in the demand for total shoulder arthroplasty (TSA) [2]. Post-surgery, there is a notable improvement in shoulder joint mobility and VAS scores compared to pre-surgery levels [3, 4]. However, pain remains a significant postoperative issue, stemming partly from the surgery itself and partly from intensified postoperative rehabilitation [5, 6]. These factors impact patient recovery, with studies by Patrick et al. [7] indicating that postoperative pain can prolong hospital stays and reduce comfort during hospitalization [8]. Additionally, studies have shown [9] an increase in morphine consumption post-surgery, sometimes exceeding recommended levels, leading to adverse reactions.

Currently, four main analgesic regimens are used clinically. Krupp et al. [10] suggest that the application of liposomal bupivacaine (LB) in TSA provides better postoperative pain control, reducing anaesthetic use and shortening medication time, thereby easing the burden on healthcare systems. However, other scholars [11] argue that local infiltration analgesia (LIA) is more effective for early post-TSA pain control. Clinically, nearly half of the analgesic approaches for shoulder arthroplasty involve single-shot interscalene block (ssISB) and continuous interscalene block (cISB) [12]. Numerous studies [13–15] indicate that interscalene block (ISB) as part of a multimodal analgesic approach significantly reduces pain scores. The choice between ssISB and cISB, however, remains debated. Bjørnholdt et al. [16] and others prefer ssISB because postoperative shoulder pain is often concentrated within the first 24 h, and a single shot of ISB suffices for pain relief without causing side effects like hoarseness, breathing difficulties, and sensory abnormalities. Conversely, another faction [17, 18] considers continuous ISB, administered through a retained nerve catheter, as the most effective method for managing moderate to severe pain following major shoulder surgery. Recent studies affirm its safety and efficacy, yet we observe that 84% of clinicians still choose not to use this pain management method [19].

In summary, there is ongoing clinical debate regarding the best analgesic approach for postoperative pain management in total shoulder arthroplasty. Therefore, this study compares different interventions and their impacts post-TSA. Specific observations include VAS pain scores at different time points, morphine consumption at various intervals and in total, and average hospital stay duration, to assess which pain management method is most effective and safe for total shoulder arthroplasty.

## Data and methods

### Literature search strategy

#### Researchers

The literature search was conducted by the second, third, and fourth authors.

#### Databases

A comprehensive search was carried out across five databases: PUBMED, Cochrane Central Register of Controlled Trials, EMBASE, Web of Science, and Scopus.

#### Search terms

The following terms were used for the search: “Total Shoulder Replacement,” “Liposomal Bupivacaine,” “Local Infiltration Analgesia,” “Single-Shot Interscalene Block,” and “Continuous Interscalene Block.”

#### Search time frame

The search covered the period from the inception of each database until November 2023.

#### Search strategy

**Fig. 1:**
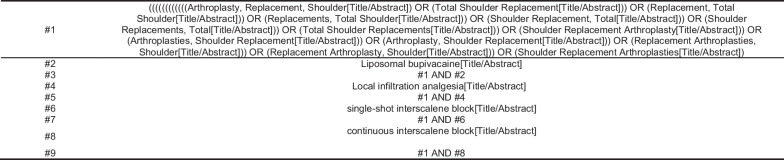
PubMed search strategy

For example, in PUBMED, the search strategy is illustrated in Fig. 1.

### Inclusion and exclusion criteria

#### Inclusion criteria

1. Studies included must involve one of the four analgesic methods under investigation in both experimental and control groups.
2. The research topic must be related to Total Shoulder Arthroplasty or Reverse Total Shoulder Arthroplasty.
3. Randomized controlled trials (RCTs).
4. Outcome measures must include at least one of the following: VAS pain scores, morphine consumption, and average hospital stay.

#### Exclusion criteria

1. Studies on shoulder surgeries other than total or reverse total shoulder arthroplasty.
2. Patients with allergies to the drugs used in the four analgesic methods, or those who cannot tolerate pain management procedures.
3. Patients unwilling to use any of the four analgesic plans.
4. Studies that are not randomized controlled trials [including non-RCTs, conference papers, reviews, meta-analyses, systematic reviews, animal studies, case reports, correspondence], or those with incomplete data or unreported findings.

### Literature screening and data extraction

The second, third, and corresponding authors utilized EndnoteX9 for screening and excluding literature. The initial screening eliminated duplicates, non-RCTs, conference papers, reviews, meta-analyses, systematic reviews, animal studies, case reports, and correspondence. The second step involved reading the abstracts of the remaining literature to determine compliance with the inclusion and exclusion criteria. In the third step, full-text reading of the remaining literature was conducted for further identification of inclusion. During this process, the three authors independently screened the literature, comparing the included studies. If there was agreement, those studies were finally included; if there were discrepancies, a discussion among all authors was held to reach a consensus. A predefined, standardized seven-item data extraction table was used to record data from the included studies, including the following headings: (1) first author of the study, (2) country, (3) year of publication, (4) study population, (5) average age, (6) gender, (7) intervention and control measures, and (8) outcome measures.

### Quality assessment of the literature

The three authors independently assessed the risk of bias in the included RCTs using the Cochrane Handbook version 5.1.0 ROB tool. The assessment covered seven aspects: (1) random sequence generation, (2) allocation concealment, (3) blinding of participants and personnel, (4) blinding of outcome assessment, (5) incomplete outcome data, (6) selective reporting, and (7) other biases. The ROB [20] was categorized into three levels: high risk (5 or more criteria), moderate risk (3 or 4 criteria), and low risk (2 or fewer criteria).

### Outcome measures

Outcome measures include VAS pain scores, morphine consumption, and average hospital stay.

### Statistical analysis

We employed Stata software (version 15.1) and followed the PRISMA NMA [21] guidelines for conducting a network meta-analysis (NMA) to compare the effectiveness of different analgesic methods. The effects of each treatment method on pain control, morphine consumption, and hospital stay were assessed by calculating the mean difference (MD) and its 95% confidence interval (CI). Additionally, an assessment of bias risk in the included studies was conducted, categorizing them into low, moderate, and high risk. Funnel plot analysis was utilized to detect possible publication bias, with results showing no evident bias. These statistical methods ensured the comprehensiveness and reliability of the analysis.

## Results

### Search results

**Fig. 2:**
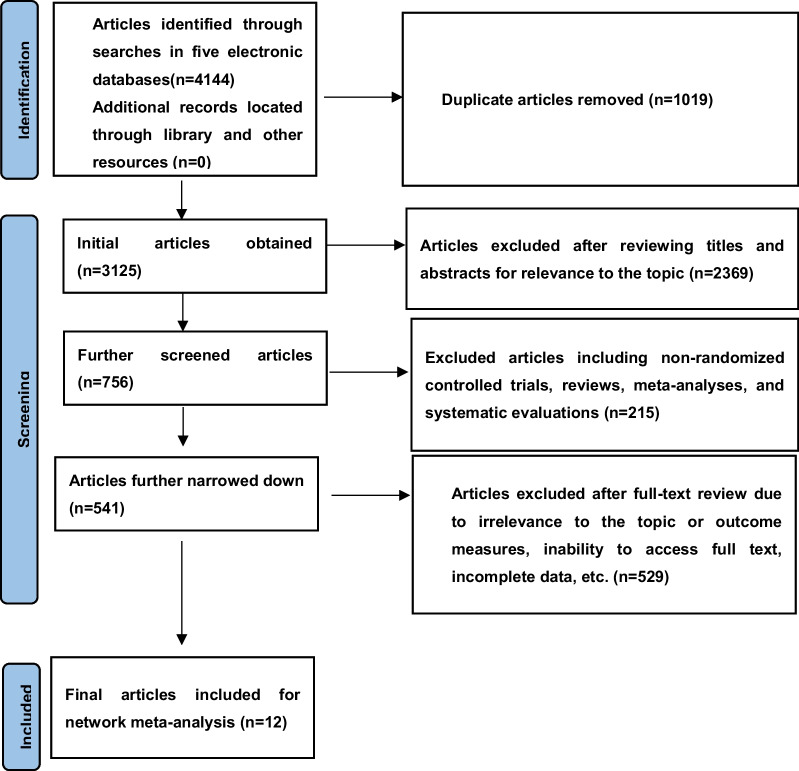
Flowchart of literature screening process

A total of 4144 articles were identified from electronic databases, with an additional 0 articles located through manual search. After removing duplicates, 3125 articles remained, and their titles and abstracts were reviewed. Of these, 2369 were excluded as they did not align with the research topic. A further 215 articles such as non-randomized controlled trials, reviews, meta-analyses, and systematic evaluations were also excluded. The remaining 541 articles underwent full-text review, leading to the exclusion of another 529 articles due to reasons including irrelevance to the topic, incompatibility with the study's outcome measures, inability to access the full text, or incomplete data. Consequently, 12 articles were ultimately included in this study. Further details are illustrated in Fig. 2.

### Literature quality evaluation results

**Fig. 3:**
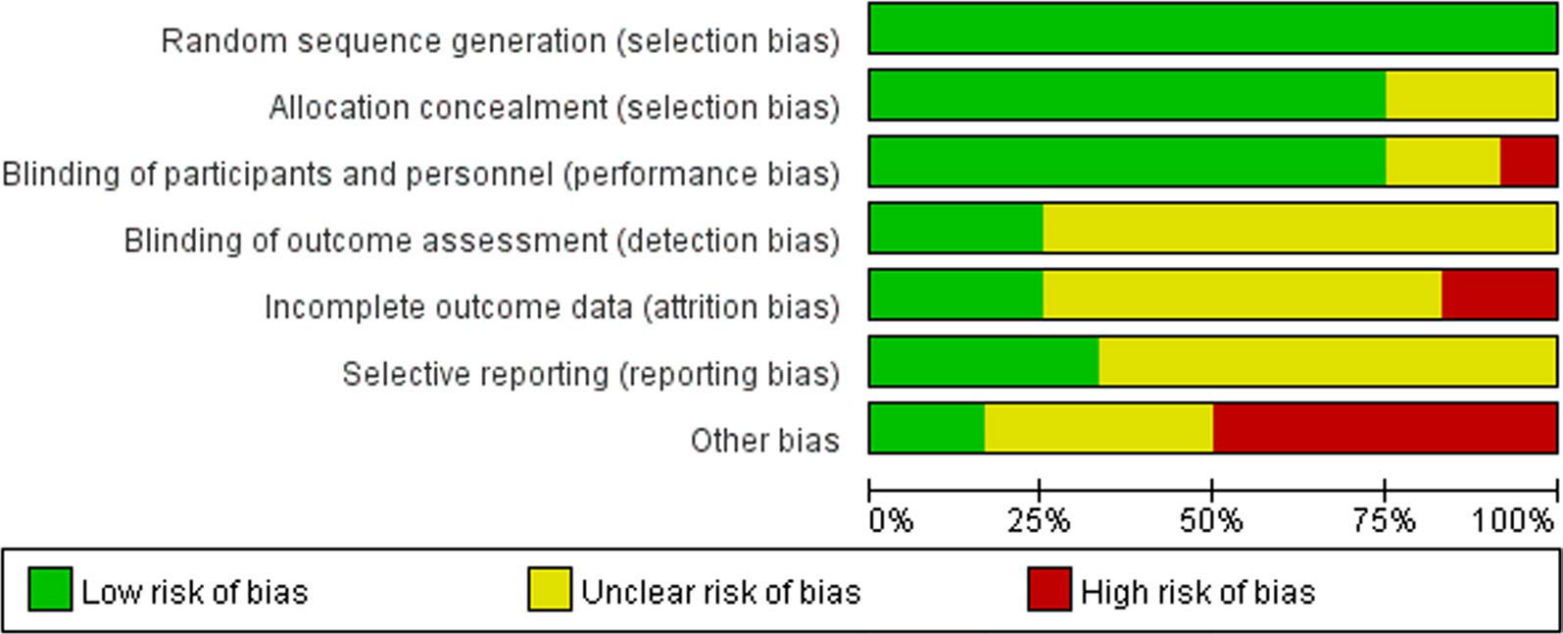
Risk of bias assessment chart 1 for included literature. Green denotes low risk of bias, yellow for unclear risk of bias, and red for high risk of bias

**Fig. 4:**
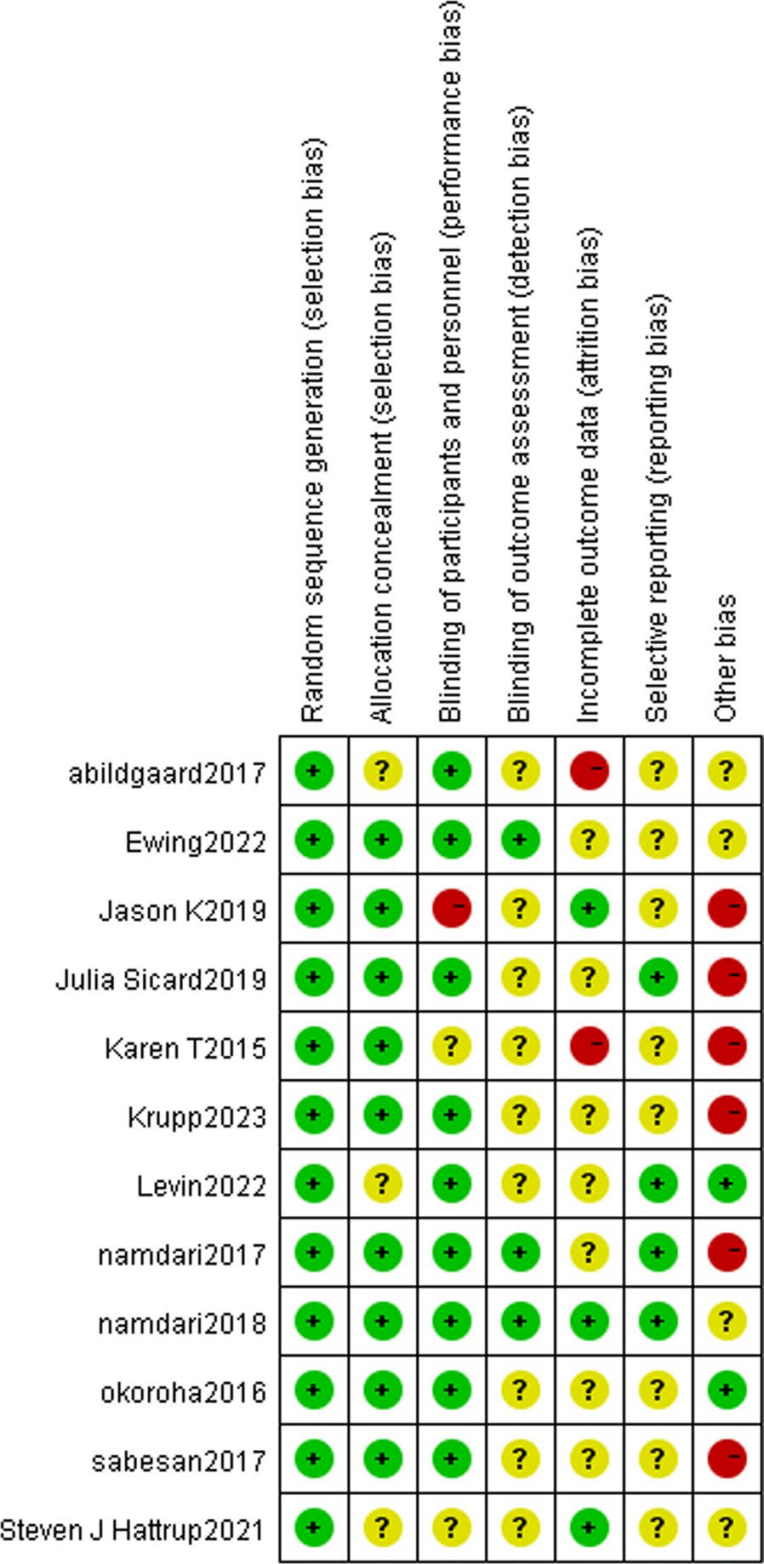
Risk of bias assessment chart 2 for included literature. Green ‘+’ denotes low risk, yellow ‘?’ for unknown risk, and red ‘−’ for high risk

Four studies were classified as low risk, one as high risk, and seven as medium risk. Among these, seven studies achieved double-blind status for both participants and observers. In contrast, four studies achieved single-blind status. Selective reporting was not mentioned in eight studies, but six exhibited a high risk of other biases. For detailed information, refer to Figs. 3 and 4. The basic characteristics of the included literature in this study are presented in Table 1.

**Table 1:** Basic characteristics of included studies

Author	Country	Year	Population	Age (Mean + SD)	Total/male/female	Intervention	Control	Outcome
Bjørnholdt et al. [16]	Denmark	2015	Patients scheduled for primary shoulder replacement	LIA: 65 ± 8; cISB: 66 ± 8	LIA: 30/15/15; cISB:31 9/22	cISB	LIA	VAS/mc/AHS
Okoroha et al. [22]	USA	2016	Patients undergoing shoulder arthroplasty	ssISB:67.1 ± 8.6; LB: 69.4 ± 8.9	ssISB:31/16/15;LB:26/12/14	ssISB	LB	VAS/mc/AHS
Abildgaard et al. [23]	Denmark	2017	Patients undergoing shoulder arthroplasty	cISB:NA LB:NA	IINB: 46/14/32; LB: 37/21/16	cISB	LB	VAS/mc/AHS
Sabesan et al. [18]	USA	2017	Patients undergoing shoulder arthroplasty	CISB: 65 ± NA; LB: 63 ± NA	cISB:36/19/17; LB:34/25/9	cISB	LB	VAS/mc/AHS
Namdari et al. [24]	USA	2017	Patients scheduled for total shoulder arthroplasty	LB:70.9 ± 9.3 ssISB:68.4 ± 8.2	LB:78/31/47; ssISB:78/40/38	ssISB	LB	VAS/mc
Namdari et al. [25]	USA	2018	Patients undergoing shoulder arthroplasty	ssISB:71.2 ± 8.6; cISB:68.6 ± 10.0	ssISB:39/24/15;cISB:39/19/20	cISB	ssISB	VAS/mc/AHS
Panchamia et al. [17]	USA	2019	Patients receiving local infiltration analgesia or interscalene block after shoulder arthroplasty	LB: 69.5 ± 8.9; ssISB: 67.8 ± 13.1; cISB: 68.1 ± 10.1	LIA:42/25/17;ssISB:42/20/22; cISB:41/19/22	cISB	ssISB, LB	VAS/mc
Sicard et al. [11]	France	2019	Patients undergoing shoulder arthroplasty	LIA: 72.2 ± 10.1; cISB: 71.7 ± 9	LIA:50/14/36; cISB: 49/21/28	cISB	LIA	VAS/mc/AHS
Hattrup et al. [26]	USA	2021	Patients undergoing shoulder arthroplasty	LB**:**69.2 ± 10.15 ssISB:70.0 ± 6.84	LB:52/28/24 ssISB:52/30/22	ssISB	LB	VAS/mc
Krupp et al. [10]	USA	2023	Patients undergoing shoulder arthroplasty	ssISB:66.9 ± NA cISB: 67.1 ± NA	ssISB:21/14/7; cISB:33/15/18	cISB	ssISB	VAS/mc/AHS
Levin et al. [27]	USA	2022	Patients undergoing primary total shoulder arthroplasty	LB:69 ± 10; cISB: 69 ± 9	LB:323/140/183; cISB:242/105/137	cISB	LB	VAS/mc
Ewing et al. [28]	USA	2022	Patients undergoing primary total shoulder arthroplasty	LIA: 70.5 ± 9.7 cISB:68.9 ± 8.5	LIA:37/28/9 cISB:37/24/13	cISB	LIA	VAS/mc/AHS

*LB* liposomal bupivacaine, *LIA* local infiltration analgesia, *ssISB* single-shot interscalene block, *cISB* continuous interscalene block, *VAS* visual analogue scale, *MC* morphine consumption, *AHS* average hospital stay, *NA* not available

### Meta-analysis results

#### VAS pain scores

##### In PACU

**Fig. 5:**
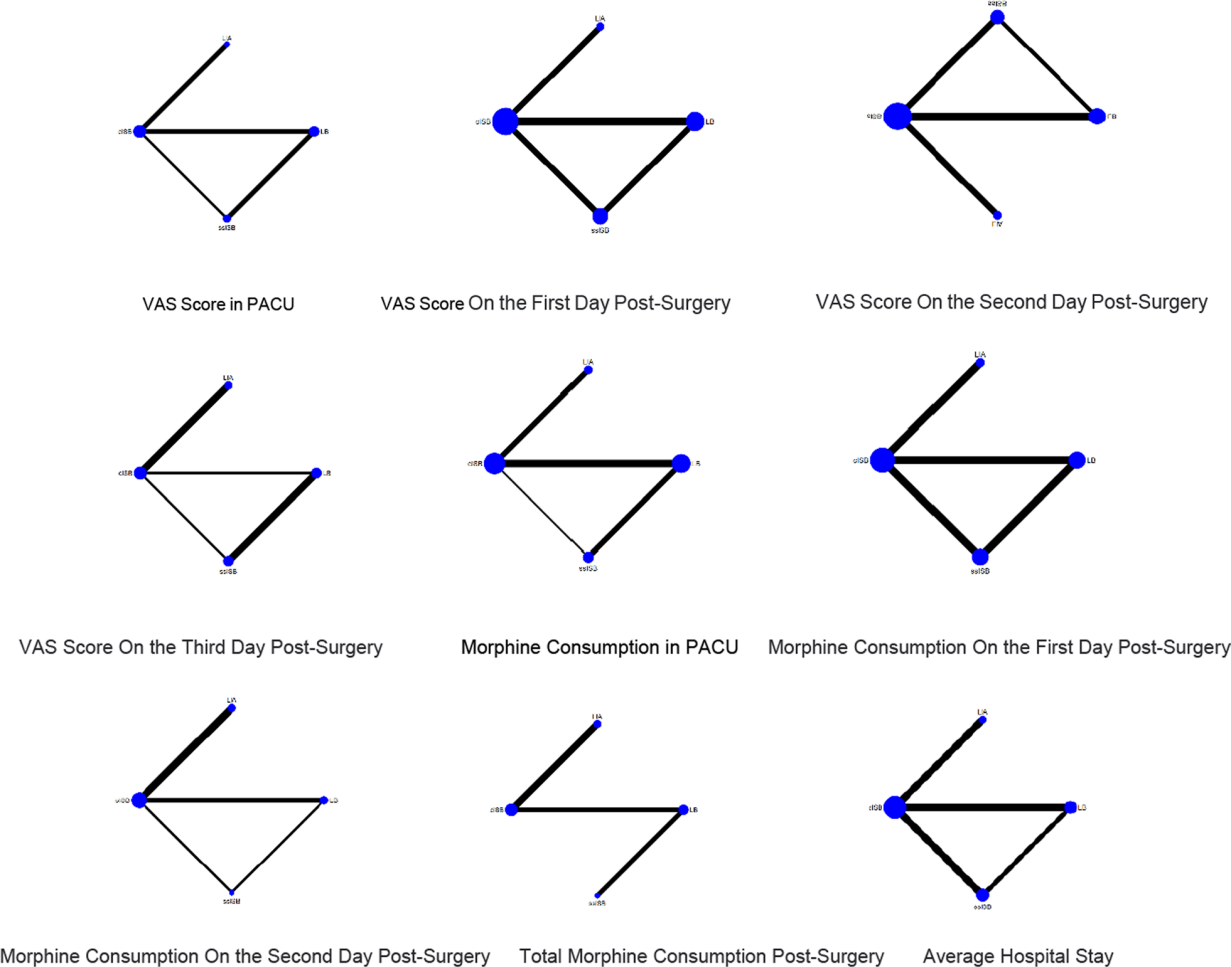
Relationship diagram of different pain relief interventions

**Fig. 6:**
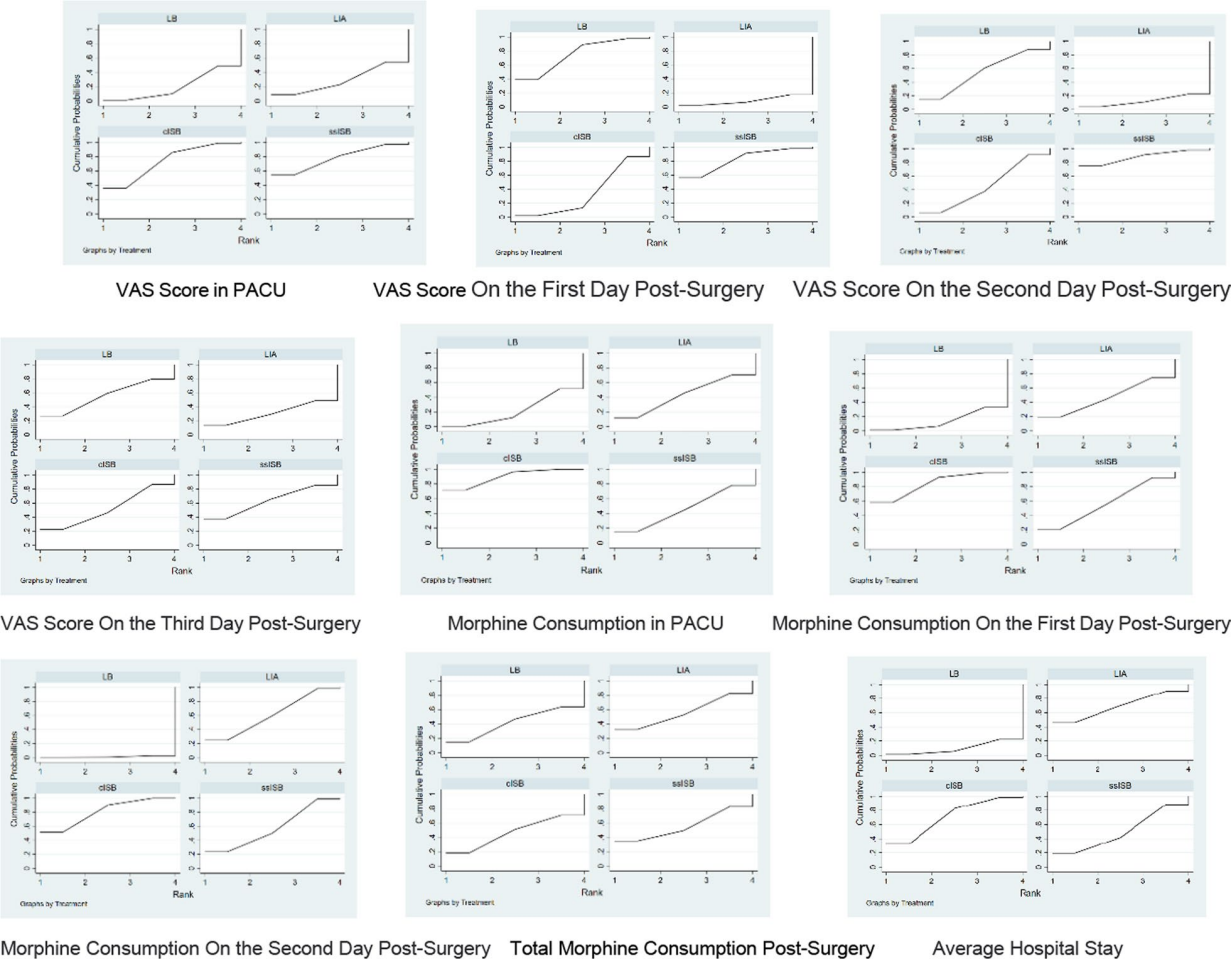
SUCRA diagram for different pain relief interventions

The network meta-analysis results showed that compared to interventions in the various experimental and control groups, the ssISB group [MD =  − 2.91, 95% CI = (− 6.44, 0.61)], cISB group [MD = -2.56, 95% CI = (− 6.09, 0.98)], and LIA group [MD =  − 0.23, 95% CI = (− 5.68, 5.21)] had superior VAS scores in the Post-Anesthesia Care Unit (PACU) compared to the control group LB. The probability ranking of different analgesic interventions in the PACU’s VAS was highest for the ssISB group (SUCRA: 77.8%, as shown in Fig. 6). The NMA chart is displayed in Fig. 5.

##### On the first day post-surgery

The results indicated that the ssISB group [MD =  − 1.41, 95% CI = (− 3.06, 0.25)], LB group [MD =  − 1.29, 95% CI = (− 2.88, 0.31)], and cISB group [MD =  − 0.67, 95% CI = (− 1.96, 0.62)] performed better on the first day post-surgery VAS compared to the LIA control group. The ssISB group ranked first in SUCRA probability ranking for this intervention (SUCRA: 82.1%, as shown in Fig. 6). The NMA chart is displayed in Fig. 5.

##### On the second day post-surgery

For the second day post-surgery, the ssISB group [MD =  − 1.17, 95% CI = (− 2.64, 0.30)], LB group [MD =  − 0.71, 95% CI = (− 2.11, 0.69)], and cISB group [MD =  − 0.60, 95% CI = (− 1.73, 0.53)] outperformed the LIA control group in terms of VAS. The ssISB group again ranked first in SUCRA (SUCRA: 88.0%, as indicated in Fig. 6). The NMA chart is presented in Fig. 5.

##### On the third day post-surgery

The meta-analysis showed that the ssISB group [MD =  − 0.56, 95% CI = (− 2.59, 1.48)], LB group [MD =  − 0.45, 95% CI = (− 2.47, 1.57)], and cISB group [MD =  − 0.32, 95% CI = (− 1.41, 0.76)] were superior on the third day post-surgery VAS compared to the LIA group. The ssISB group was again ranked first in SUCRA (SUCRA: 63.0%, as shown in Fig. 6). The NMA chart is displayed in Fig. 5.

#### Morphine consumption

##### In PACU

Relative to the various experimental and control group interventions, the cISB group [MD =  − 27.39, 95% CI = (− 53.78, − 1.00)], ssISB group [MD =  − 8.84, 95% CI = (− 39.70, 22.02)], and LIA group [MD =  − 8.75, 95% CI = (− 49.92, 32.42)] showed reduced morphine consumption in the PACU compared to the LB control group. The cISB group ranked first in the SUCRA probability ranking for this intervention (SUCRA: 89.2%, as shown in Fig. 6). The NMA chart is shown in Fig. 5.

##### On the first day post-surgery

The cISB group [MD =  − 8.17, 95% CI = (− 16.63, 0.28)], ssISB group [MD =  − 5.23, 95% CI = (− 13.54, 3.08)], and LIA group [MD =  − 4.15, 95% CI = (− 17.18, 8.87)] demonstrated lower morphine consumption on the first day post-surgery compared to the LB group. The cISB group ranked first in the SUCRA probability ranking (SUCRA: 83.6%, as indicated in Fig. 6). The NMA chart is presented in Fig. 5.

##### On the second day post-surgery

For the second day post-surgery, the cISB group [MD =  − 14.85, 95% CI = (− 24.60, − 5.09)], LIA group [MD = -12.71, 95% CI = (− 24.60, − 0.81)], and ssISB group [MD =  − 12.09, 95% CI = (− 21.96, − 2.21)] were more effective compared to the LB group. The cISB group ranked first in SUCRA (SUCRA: 80.5%, as shown in Fig. 6). The NMA chart is displayed in Fig. 5.

##### Total morphine consumption post-surgery

The LIA group [MD =  − 0.97, 95% CI = (− 23.67, 21.73)], ssISB group [MD =  − 0.65, 95% CI = (− 3.31, 2.01)], and cISB group [MD =  − 0.81, 95% CI = (− 23.48, 21.87)] exhibited lower total morphine consumption post-surgery compared to the LB group. The LIA group ranked first in SUCRA (SUCRA: 55.9%, as indicated in Fig. 6). The NMA chart is shown in Fig. 5.

#### Average hospital stay

The meta-analysis revealed that the cISB group [MD =  − 1.03, 95% CI = (− 2.21, 0.14)], LIA group [MD =  − 1.07, 95% CI = (− 2.80, 0.66)], and ssISB group [MD =  − 0.71, 95% CI = (− 1.99, 0.56)] had shorter average hospital stays compared to the LB group. The cISB group was ranked first in SUCRA (SUCRA: 71.2%, as shown in Fig. 6). The NMA chart will be displayed in Fig. 5.

#### Publication bias analysis

**Fig. 7:**
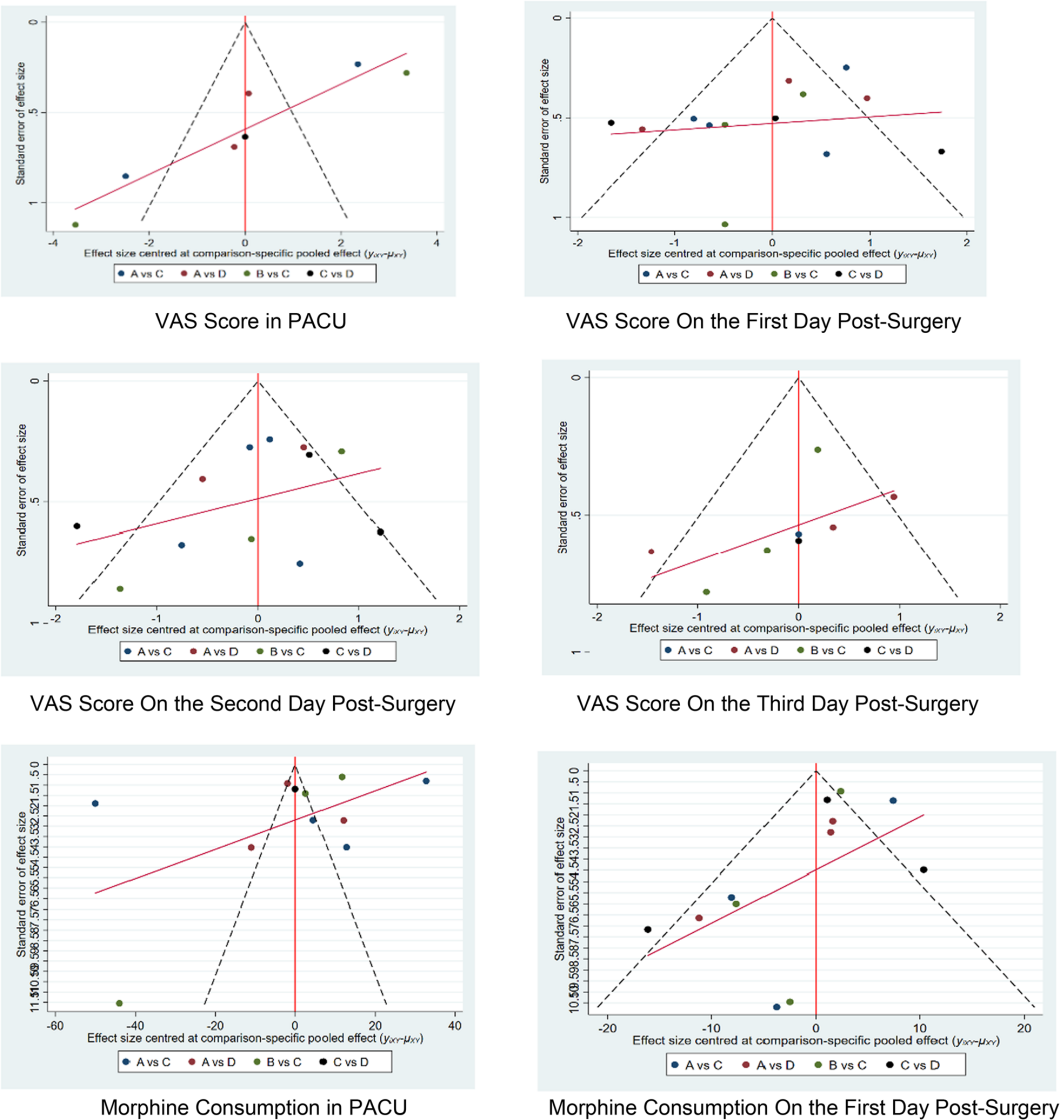

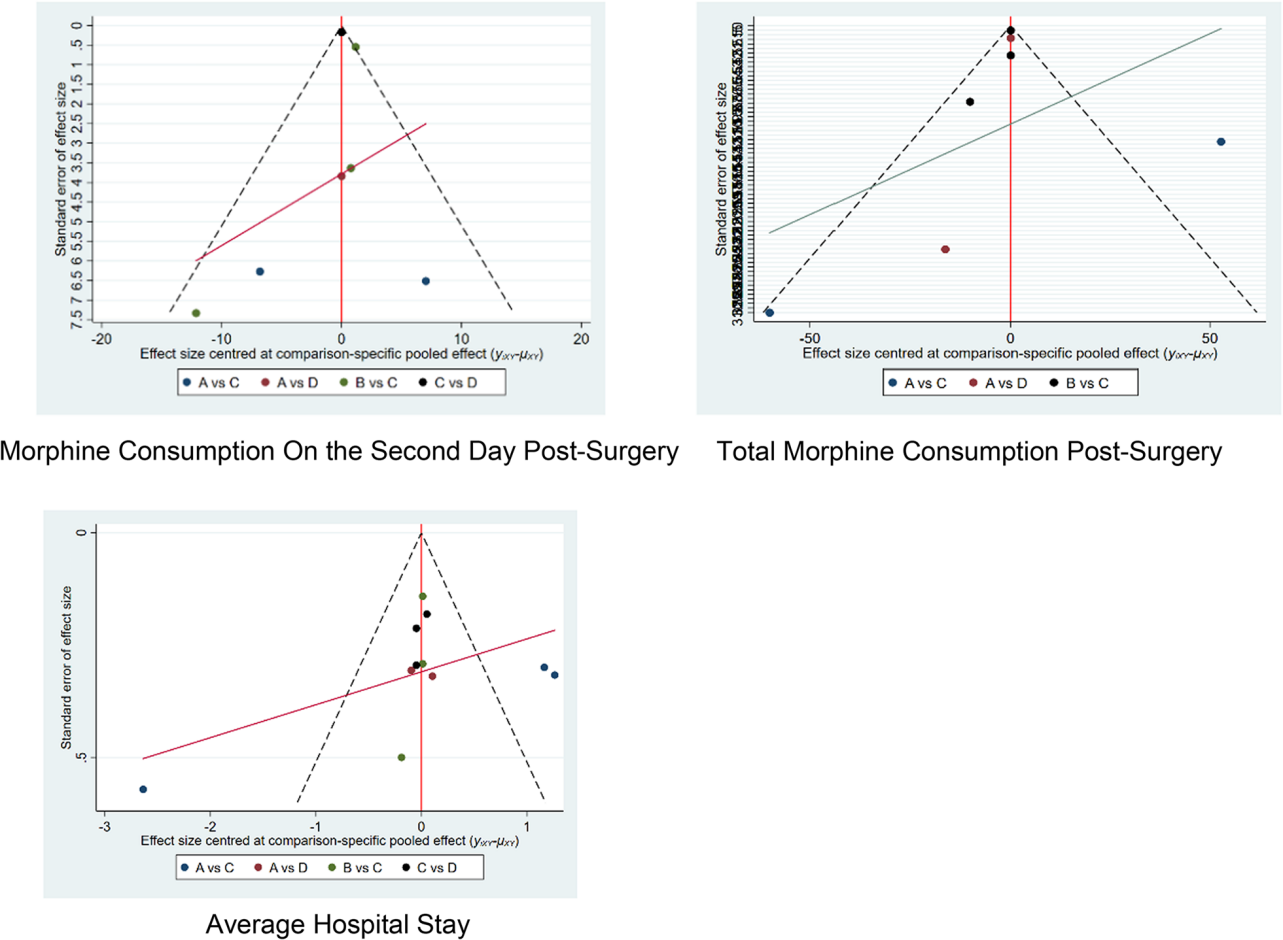
Publication bias analysis of different pain relief methods

We constructed individual funnel plots for nine outcomes across three sets of indicators to examine the presence of publication bias. No apparent publication bias was observed in the nine funnel plots. Detailed information can be found in the table below (Table 2, Fig. 7).

**Table 2:** League table of different pain relief interventions

VAS score in PACU			
_D_	_C_	_B_	_A_
D	0.36 (− 3.72,4.44)	2.68 (− 3.14,8.50)	2.91 (− 0.61,6.44)
− 0.36 (− 4.44,3.72)	C	2.32 (− 1.83,6.47)	2.56 (− 0.98,6.09)
− 2.68 (− 8.50,3.14)	− 2.32 (− 6.47,1.83)	B	0.23 (− 5.21,5.68)
− 2.91 (− 6.44,0.61)	− 2.56 (− 6.09,0.98)	− 0.23 (− 5.68,5.21)	A

VAS score on the first day post-surgery			
_D_	_A_	_C_	_B_
D	0.12 (− 0.89,1.13)	0.74 (− 0.30,1.77)	1.41 (− 0.25,3.06)
− 0.12 (− 1.13,0.89)	A	0.62 (− 0.32,1.56)	1.29 (− 0.31,2.88)
− 0.74 (− 1.77,0.30)	− 0.62 (− 1.56,0.32)	C	0.67 (− 0.62,1.96)
− 1.41 (− 3.06,0.25)	− 1.29 (− 2.88,0.31)	− 0.67 (− 1.96,0.62)	B

VAS score on the second day post-surgery			
_D_	_A_	_C_	_B_
D	0.46 (− 0.52,1.44)	0.57 (− 0.36,1.50)	1.17 (− 0.30,2.64)
− 0.46 (− 1.44,0.52)	A	0.12 (− 0.72,0.95)	0.71 (− 0.69,2.11)
− 0.57 (− 1.50,0.36)	− 0.12 (− 0.95,0.72)	C	0.60 (− 0.53,1.73)
− 1.17 (− 2.64,0.30)	− 0.71 (− 2.11,0.69)	− 0.60 (− 1.73,0.53)	B

VAS score on the third day post-surgery			
_D_	_A_	_C_	_B_
D	0.11 (− 0.97,1.18)	0.23 (− 1.48,1.95)	0.56 (− 1.48,2.59)
− 0.11 (− 1.18,0.97)	A	0.13 (− 1.58,1.83)	0.45 (− 1.57,2.47)
− 0.23 (− 1.95,1.48)	− 0.13 (− 1.83,1.58)	C	0.32 (− 0.76,1.41)
− 0.56 (− 2.59,1.48)	− 0.45 (− 2.47,1.57)	− 0.32 (− 1.41,0.76)	B

Morphine consumption in PACU			
_C_	_D_	_B_	_A_
C	18.55 (− 18.40,55.51)	18.64 (− 13.81,51.10)	27.39 (1.00,53.78)
− 18.55 (− 55.51,18.40)	D	0.09 (− 48.64,48.82)	8.84 (− 22.02,39.70)
− 18.64 (− 51.10,13.81)	− 0.09 (− 48.82,48.64)	B	8.75 (− 32.42,49.92)
− 27.39 (− 53.78, − 1.00)	− 8.84 (− 39.70,22.02)	− 8.75 (− 49.92,32.42)	A

Morphine consumption on the first day post-surgery			
_C_	_D_	_B_	_A_
C	2.94 (− 5.16,11.05)	4.02 (− 6.12,14.16)	8.17 (− 0.28,16.63)
− 2.94 (− 11.05,5.16)	D	1.08 (− 12.01,14.16)	5.23 (− 3.08,13.54)
− 4.02 (− 14.16,6.12)	− 1.08 (− 14.16,12.01)	B	4.15 (− 8.87,17.18)
− 8.17 (− 16.63,0.28)	− 5.23 (− 13.54,3.08)	− 4.15 (− 17.18,8.87)	A

Morphine consumption on the second day post-surgery			
_C_	_B_	_D_	_A_
C	2.14 (− 5.68,9.96)	2.76 (− 6.60,12.12)	14.85 (5.09,24.60)
− 2.14 (− 9.96,5.68)	B	0.62 (− 10.89,12.13)	12.71 (0.81,24.60)
− 2.76 (− 12.12,6.60)	− 0.62 (− 12.13,10.89)	D	12.09 (2.21,21.96)
− 14.85 (− 24.60, − 5.09)	− 12.71 (− 24.60, − 0.81)	− 12.09 (− 21.96, − 2.21)	A

Total morphine consumption post-surgery			
_B_	_D_	_C_	_A_
B	0.32 (− 22.53,23.17)	0.16 (− 0.85,1.17)	0.97 (− 21.73,23.67)
− 0.32 (− 23.17,22.53)	D	− 0.16 (− 22.99,22.67)	0.65 (− 2.01,3.31)
− 0.16 (− 1.17,0.85)	0.16 (− 22.67,22.99)	C	0.81 (− 21.87,23.48)
− 0.97 (− 23.67,21.73)	− 0.65 (− 3.31,2.01)	− 0.81 (− 23.48,21.87)	A

Average hospital stay			
_C_	_B_	_D_	_A_
C	− 0.04 (− 1.31,1.24)	0.32 (− 0.81,1.45)	1.03 (− 0.14,2.21)
0.04 (− 1.24,1.31)	B	0.36 (− 1.35,2.06)	1.07 (− 0.66,2.80)
− 0.32 (− 1.45,0.81)	− 0.36 (− 2.06,1.35)	D	0.71 (− 0.56,1.99)
− 1.03 (− 2.21,0.14)	− 1.07 (− 2.80,0.66)	− 0.71 (− 1.99,0.56)	A

D: ssISB; C: cISB; B: LIA; A: LB

## Discussion

In this study, we compared the analgesic efficacy of various pain relief methods following total shoulder arthroplasty. A total of 12 randomized controlled trials were included, encompassing four different analgesic techniques and involving 1537 patients who had undergone total shoulder arthroplasty, representing a considerably large sample size. Notably, the ssISB method showed superior performance in controlling early postoperative pain, especially in the PACU and initial postoperative stages. Additionally, the cISB method stood out in reducing postoperative morphine consumption and shortening the average hospital stay. While the LIA method ranked first in reducing total morphine consumption, it was less effective in pain control. The LB method was less satisfactory in most assessment criteria. These findings underscore the importance of selecting appropriate pain management strategies for different postoperative recovery phases, offering valuable insights for clinicians to optimize postoperative pain management.

Previous studies have indicated that the duration of shoulder arthroplasty and the extent of soft tissue damage correlate with increased pain mediators due to surgical procedures, including rotator cuff repair and prosthesis implantation [29]. This correlation is directly related to postoperative pain, which is less pronounced in hemiarthroplasty compared to total shoulder arthroplasty, as reflected in the VAS scores [3]. The first 48 h post-surgery is considered the most painful period by many scholars [14, 30], a finding corroborated by this study. The ssISB group exhibited significant differences in pain relief in the recovery room and at 24 and 48 h, stabilizing at 72 h.

Morphine consumption is inversely related to VAS scores. While opiates remain the best pain relief during the perioperative period of shoulder arthroplasty, their dependency and addictive properties are well-known. Overuse can be more harmful than beneficial in the long term. Therefore, reducing morphine use has become a goal in assessing the efficacy of pain relief methods. Recent studies over the past decade have explored various analgesic techniques, noting the peak of postoperative pain within the first 24 h following shoulder arthroplasty. Extensive research [22, 27, 31, 32] has shown that ssISB can significantly alleviate intraoperative and postoperative pain, reducing morphine consumption by more than half. However, as the analgesic effect diminishes with drug metabolism, rebound pain after 24 h can adversely affect postoperative recovery. Furthermore, studies by Vorobeichik [30] and others have shown that patients with poor pain control within 48 h have only about a 50% chance of achieving satisfactory long-term pain relief. These issues, including pain rebound and difficulty in maintaining effective analgesia, led Matthew [8] and colleagues to attempt continuous blockade through catheter placement, proving that cISB can offer better pain relief and less morphine use. This aligns with our study's findings of lower morphine use within 48 h post-surgery. However, our study revealed that the lowest overall postoperative morphine consumption was not in the cISB group but in the LB group. This is because liposomal bupivacaine infiltration is a gradual and sustained release process that effectively prolongs the duration of anaesthetic effect with minimal rebound pain and complications [10, 24, 27].

In this study, the average hospital stay recorded was relatively short, attributable to patients readmitted for pain management after total shoulder arthroplasty (TSA), rather than the entire duration of hospital stay for the shoulder replacement surgery. Previous studies have shown that continuous interscalene analgesia (CISA) significantly reduces the time required for discharge preparation after TSA, corroborating the findings of our study. CISA, by providing sustained and effective analgesia, facilitates greater passive shoulder mobility, obviates the need for intravenous opioid administration, and does not damage nerves, with no significant complications. It offers optimal pain relief in the first 24 h, reducing morphine consumption and shortening hospital stays [33, 34]. In our study, the liposomal bupivacaine (LB) group also performed well in terms of average hospital stay, as it too involves a continuous release process. Previous studies indicate that LB reduces pain, enhances patient satisfaction, minimizes complications, and accelerates discharge [18, 22].

In conclusion, our study demonstrates that ssISB is most effective in postoperative pain control, while cISB excels in reducing morphine consumption and shortening hospital stay. By contrast, LIA is most effective in reducing total morphine consumption but is less effective in pain control. The LB group showed generally poor performance across all parameters. These results provide crucial guidance for the selection of appropriate pain management strategies in clinical practice. However, as this study is a meta-analysis and a secondary research involving only 12 randomized controlled trials, it has significant limitations. Future research, involving higher quality randomized controlled trials, is needed to further corroborate these findings.

## Data Availability

Being a meta-analysis, this study did not require patient consent. The data are both credible and reliable.
